# Influence of Thread Geometry and Bone Density on Stress Distribution in Dental Implants: A Finite Element Study

**DOI:** 10.7759/cureus.93413

**Published:** 2025-09-28

**Authors:** Vinay Rana, Swatantra Agarwal, Reena Mittal, Subhra Rout, Medha Upadhyay, Shubham Prince, L Suzane, Chetna Saini, Seema Gupta

**Affiliations:** 1 Department of Prosthodontics, Kothiwal Dental College and Research Centre, Moradabad, IND; 2 Department of Orthodontics, Kothiwal Dental College and Research Centre, Moradabad, IND

**Keywords:** bone density, dental implants, finite element analysis, stress distribution, thread design

## Abstract

Introduction: Dental implant success relies on optimal stress distribution at the implant-bone interface, which may be influenced by thread design and bone density. While V-shaped and buttress threads have been extensively studied, square and trapezoidal designs remain underexplored, particularly in varying bone densities. This study aimed to compare the stress distribution patterns of square and trapezoidal thread designs using three-dimensional (3D) finite element analysis (FEA).

Materials and methods: Eight 3D finite element models were constructed by combining two thread designs (square and trapezoidal) with four bone densities. A mandibular first molar implant (12 mm length and 4.5 mm diameter) was modeled in SolidWorks (Dassault Systèmes SolidWorks Corp., Waltham, MA), and mesh convergence was validated. The material properties were assigned assuming linear elasticity and isotropy. A 100 Newtons (N) axial load was applied to the crown, and von Mises stresses were analyzed in the implant, cortical bone, and cancellous bone in megapascals (MPa) using Ansys software (Ansys, Inc., Canonsburg, PA).

Results: Trapezoidal threads generated higher implant stresses (7.16-18.83 MPa) than square threads (5.21-15.08 MPa), particularly in low-density bone. Square threads transferred more stress to the cortical bone (3.69-9.60 MPa vs. trapezoidal: 2.73-7.98 MPa) across all densities. In the cancellous bone, the trapezoidal threads induced higher stresses in the dense bone but converged with the square threads in the low-density bone. Stress concentrations were localized at the thread crests in dense bone but were dispersed diffusely in low-density bone.

Conclusion: The thread design significantly affected stress distribution, with square threads reducing implant stress but increasing cortical bone load, whereas trapezoidal threads minimized cortical stress at the cost of higher implant stress. Square threads may be preferable for dense bones, whereas trapezoidal designs may enhance stability in low-density bones. Preoperative bone density assessment is critical for optimizing thread selection and ensuring long-term implant success.

## Introduction

Dental implants have revolutionized the treatment of partial and complete edentulism, offering superior outcomes compared with traditional fixed or removable partial dentures. Implants provide enhanced aesthetics, comfort, speech, and preservation of oral hard and soft tissues, making them the preferred choice for tooth replacement [1]. The long-term success of dental implants depends on their ability to effectively transfer occlusal forces to the surrounding bone while maintaining stability and preventing bone resorption [2]. This biomechanical interaction is influenced by several factors, including implant geometry, thread design, surface characteristics, loading conditions, and bone quality [3]. Among these, implant thread design plays a pivotal role in determining the stress distribution at the implant-bone interface, directly affecting primary stability and osseointegration [4].

Implant thread designs, such as V-shaped, square, buttress, and reverse buttress, significantly affect the mechanical and biological fixation of implants [5]. Mechanical fixation, achieved through the interaction of implant threads with the bone, is critical for initial stability, whereas biological fixation via osseointegration ensures long-term implant success [6,7]. The design of the threads influences the contact area between the implant and bone, which is a key determinant of osseointegration strength [4-7]. Additionally, bone density (classified as D1 to D4, ranging from dense cortical to porous cancellous bone) profoundly affects the load-bearing capacity of the implant-bone interface. Variations in bone density can alter stress distribution patterns, making it essential to evaluate thread designs across different bone types to optimize clinical outcomes [8].

Finite element analysis (FEA), a sophisticated computational tool, has been widely employed since the 1970s to study stress distribution in dental implants [4,5]. FEA allows for the simulation of complex biomechanical systems by discretizing structures into elements and nodes, enabling the precise analysis of stress and strain under various loading conditions. In particular, three-dimensional (3D) FEA offers a detailed representation of the implant-bone interface, surpassing the limitations of two-dimensional models. By integrating data from imaging techniques such as cone-beam computed tomography (CBCT), which provides accurate internal geometry with reduced radiation exposure, 3D FEA facilitates reliable predictions of implant performance without the risks and costs associated with experimental implantation [9].

Although V-shaped and reverse buttress thread designs have been studied, square and trapezoidal thread designs have received less attention, particularly in the context of varying bone densities [5]. These designs hold promise for improving the load distribution and stability, especially in patients with compromised bone quality. This study addressed this gap by investigating the stress distribution patterns of square and trapezoidal thread designs across four bone densities (D1-D4) using 3D FEA. By exploring these under-examined thread geometries and their biomechanical behavior in diverse bone types, this study aimed to provide novel insights into optimizing implant designs for enhanced clinical success, particularly in challenging cases involving poor bone quality.

## Materials and methods

Study design and setting

This study was designed as a 3D FEA that utilized computational modeling to simulate biomechanical interactions at the implant-bone interface, focusing on stress distribution patterns in the cortical and cancellous bones under controlled loading conditions. This study adopted a comparative approach by analyzing eight distinct finite element models representing combinations of two thread designs and four bone densities. This study was conducted at the Department of Prosthodontics, Kothiwal Dental College and Research Centre, Moradabad, Uttar Pradesh, India. The study was conducted over 12 months, from January 2024 to December 2024, encompassing data acquisition, model construction, FEA, and data evaluation. This study utilized computer-based FEA and model analysis. All simulations were performed on digitally generated geometric models and did not involve human subjects, animal experiments, or biological samples. Therefore, ethical approval from an institutional review board was not required for this study.

Construction of the bone model

For this study, CBCT scan files available in the database were retrieved to represent a patient’s mandible with a missing first molar. The Digital Imaging and Communications in Medicine (DICOM) file was converted to a stereolithography (STL) format (RadiAnt DICOM viewer, version 2024.2, Medixant, Poznań, Poland). A bone block model representing the mandibular first molar region was constructed with dimensions of 20 mm in height and 12 mm in width. The model consisted of a cancellous core surrounded by a 2 mm layer of cortical bone. The bone block was modeled using SolidWorks (version 25, Dassault Systèmes SolidWorks Corp., Waltham, MA).

Construction of the implant model

Two 3D cylindrical implant finite element models, each with a length of 12 mm and a diameter of 4.5 mm, were designed with square and trapezoidal thread configurations. The thread designs featured a pitch of 0.8 mm, thread inclination of 45°, and thread width of 0.4 mm. Additionally, an abutment (4 mm in diameter, 5 mm high, 2 mm high, and 50 mm divergent occlusally) and a porcelain crown of the mandibular first molar were constructed with a mesiodistal width of 11 mm, buccolingual width of 10 mm, and occlusocervical height of 7 mm. Eight models were created to represent combinations of two thread designs (square and trapezoidal) across four bone densities (D1, D2, D3, and D4) classified by Misch [10]. All the modeling was performed using SolidWorks.

Mesh generation

The geometric models were converted into finite element models using HyperMesh (version 10.0, Altair Engineering, Inc., Troy, MI), a general-purpose pre-processor compatible with FEA software. The models were discretized into a large number of elements and nodes to ensure an accurate representation of complex geometries. A tetrahedral mesh was employed because of its suitability for irregular geometries, such as mandible and implant structures. The mesh details, including the number of elements and nodes in each model, are listed in Table 1.

**Table 1:** Mesh details of each model.

Models	Component	Number of nodes	Number of elements
1	Crown	6703	3967
2	Cortical bone	433770	28254
3	Cancellous bone	61499	41176
4	Fixture with square thread	19293	11470
5	Fixture with trapezoidal thread	18749	11590
6	Abutment and screw assembly	2044	1215

Mesh convergence analysis

A mesh convergence analysis was performed to ensure the reliability and accuracy of the FEA results. The mesh density was incrementally increased by refining the element size in critical regions, such as the implant-bone interface and thread regions, until the variation in von Mises stress values between successive mesh refinements was less than 5%. This process involved generating multiple mesh configurations with element sizes ranging from coarse (approximately 0.5 mm) to fine (approximately 0.1 mm) in high-stress areas. The convergence criterion ensured that further mesh refinement did not significantly alter the stress distribution results, thereby balancing the computational efficiency and accuracy. The final mesh configuration was selected based on this analysis to optimize the computational resources while maintaining the precision.

Assigning material properties

All materials in the finite element models were assumed to be homogeneous, linearly elastic, and isotropic in nature. The material properties, including the Young’s modulus (modulus of elasticity) and Poisson’s ratio, were assigned based on values derived from the literature (Table 2) [8]. These properties were applied to the cortical bone, cancellous bone (with variations in D1-D4 densities), implants (titanium), abutments, and porcelain crowns to accurately simulate their biomechanical behavior.

**Table 2:** Material properties used in the study. GPa: gigapascals. D1 bone denotes dense cortical bone; D2 bone denotes cortical/trabecular bone; D3 bone denotes thin cortical/fine trabecular bone; D4 bone denotes fine trabecular bone.

Materials	Young’s modulus (GPa)	Poisson’s ratio
Porcelain crown	82.8	0.3
Ceramic crown	82.8	0.3
Titanium abutment	110	0.3
Titanium implant	110	0.3
Titanium screw	110	0.3
D1 bone	9.5	0.3
D2 bone	5.5	0.3
D3 bone	1.6	0.3
D4 bone	0.69	0.3

Applying boundary conditions

Boundary conditions were applied to constrain the finite element models in all directions, preventing the rotation or movement of the cortical and cancellous bone at the periphery of the model. This was achieved by fixing nodes at the base and lateral surfaces of the bone block to simulate a stable mandibular structure.

Load application and execution of the analysis

A static load of 100 Newtons (N) was applied at a 0° angle to the long axis of the implant, which was positioned at the center of the central fossa of the porcelain crown. FEA was performed using Ansys software (version 25 R2, Ansys, Inc., Canonsburg, PA). von Mises stress maps were generated to analyze the stress distribution in the implant, cortical bone, and cancellous bone across all eight models.

The primary outcome assessed was the pattern of stress distribution (von Mises stresses) in the implant, cortical bone, and cancellous bone for each of the eight models (two thread designs across four bone densities). The stress distribution patterns were evaluated to compare the biomechanical performance of square and trapezoidal thread designs for different bone densities (D1-D4), determine the influence of thread design on load distribution and implant stability in varying bone qualities, and identify the thread design that optimizes stress distribution and enhances implant success, particularly in patients with poor bone quality (e.g., D4 bone). The von Mises stress data were qualitatively assessed in megapascals (MPa) to observe the differences in stress concentration and distribution.

## Results

The analysis in Table 3 shows that the maximum stresses in the implant, cortical bone, and cancellous bone varied significantly with the thread type and bone density. For square thread designs, as bone density decreased from D1 to D4, the stress at the implant increased from 5.21 MPa to 15.08 MPa, the stress at the cortical bone increased from 3.69 MPa to 9.60 MPa, and the stress at the cancellous bone decreased from 1.79 MPa to 1.03 MPa. In contrast, trapezoidal thread designs exhibited higher implant stresses, ranging from 7.16 MPa (D1) to 18.83 MPa (D4), with cortical bone stresses increasing from 2.73 MPa to 7.98 MPa and cancellous bone stresses decreasing from 1.98 MPa to 1.09 MPa. Overall, trapezoidal threads consistently induced higher stresses in the implant and lower stresses in the cortical bone than square threads, while cancellous bone stresses were generally higher in trapezoidal threads at higher densities (D1, D2) but converged with square threads at lower densities (D4).

**Table 3:** von Mises stress (MPa) in implant body, cancellous bone, and cortical bone, with square and trapezoidal thread designs in different bone densities from D1 to D4. Values represent von Mises stresses in megapascals (MPa) from the finite element analysis (FEA) study. Statistical parameters (e.g., N, %, mean ± SD) are not applicable. D1 bone denotes dense cortical bone; D2 bone denotes cortical/trabecular bone; D3 bone denotes thin cortical/fine trabecular bone; D4 bone denotes fine trabecular bone.

Bone quality	Implant		Cancellous		Cortical	
	Square	Trapezoidal	Square	Trapezoidal	Square	Trapezoidal
D1	5.21	7.16	3.69	2.73	1.79	1.98
D2	8.87	10.90	3.95	3.05	1.75	1.97
D3	12.03	17.49	6.16	4.82	1.34	1.52
D4	15.08	18.83	9.60	7.98	1.03	1.09

**Figure 1:**
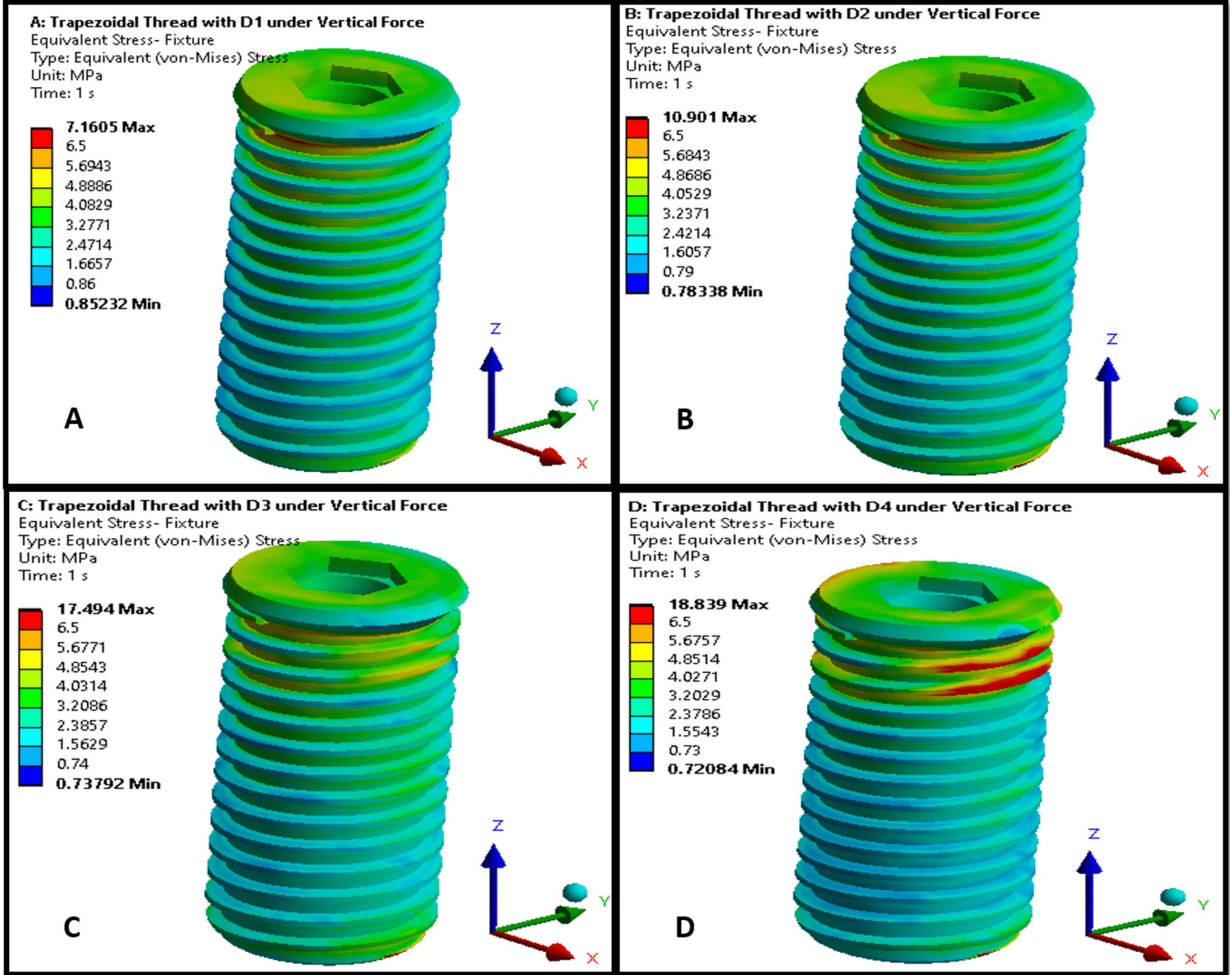
von Mises stress distribution on a trapezoidal-threaded implant in different bone densities: (A) D1 bone (dense cortical); (B) D2 bone (cortical/trabecular); (C) D3 bone (thin cortical/fine trabecular); (D) D4 bone (fine trabecular). Colors indicate von Mises stress in megapascals (MPa): blue (low stresses), green (moderate), yellow (high), and red (critical). Arrows denote anatomical/loading directions. This figure is generated using Ansys software for finite element modeling of stress distribution.

**Figure 2:**
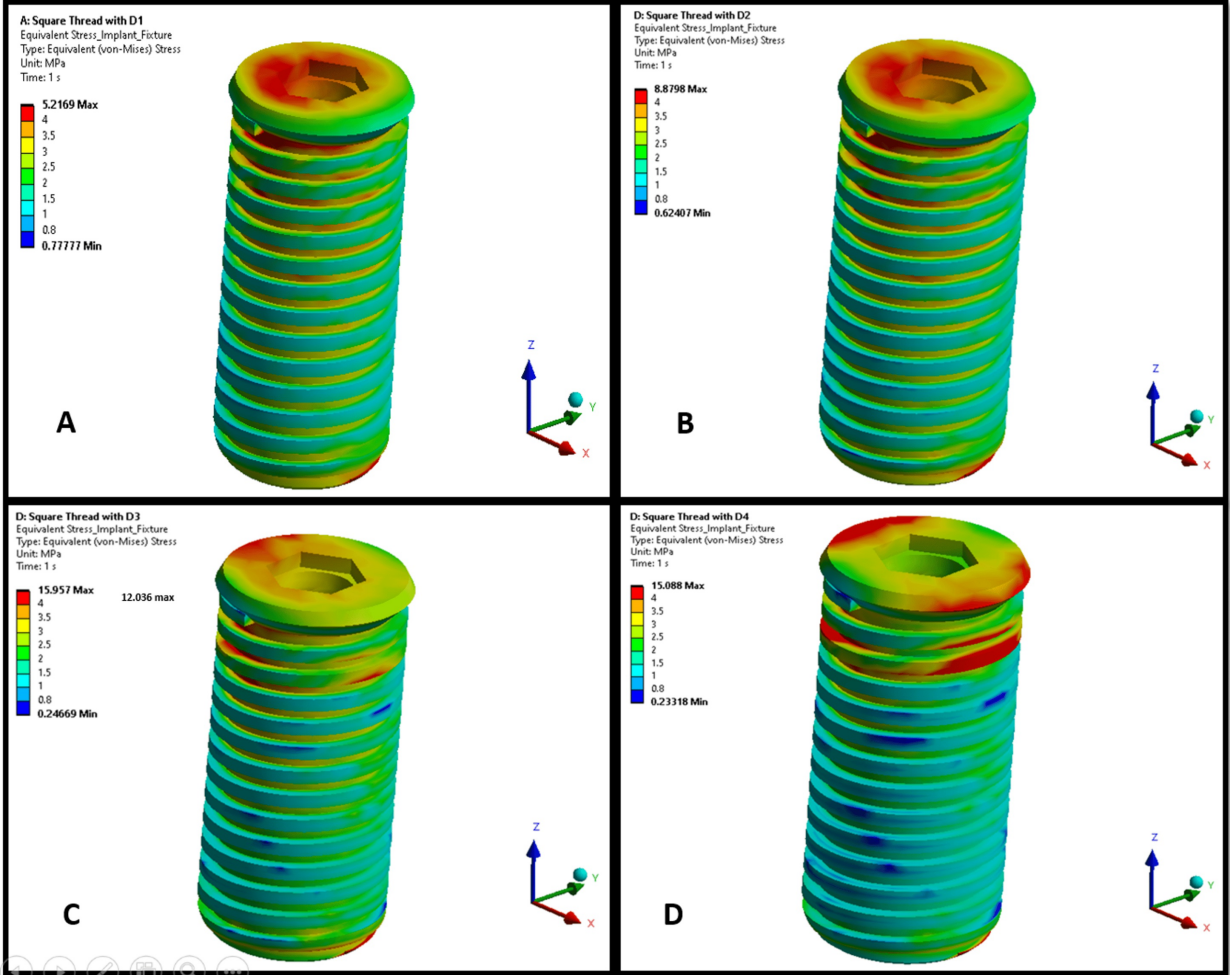
von Mises stress distribution on a square-threaded implant in different bone densities: (A) D1 bone (dense cortical), (B) D2 bone (cortical/trabecular), (C) D3 bone (thin cortical/fine trabecular), and (D) D4 bone (fine trabecular). Colors indicate von Mises stress in megapascals (MPa): blue (low stresses), green (moderate), yellow (high), and red (critical). Arrows denote anatomical/loading directions. This figure is generated using Ansys software for finite element modeling of stress distribution.

Figures 1, 2 illustrate the maximum von Mises stress distributions in an implant body across bone densities D1-D4. Both thread designs showed a similar trend of increasing stress levels in the implant as bone density decreased from D1 to D4, with stress concentrations primarily around the threaded regions, particularly at the thread crests and roots. In lower-density bones (D3 and D4), stresses were more dispersed across the implant for both thread types, indicating greater strain owing to reduced bone support. In higher-density bones (D1 and D2), the stresses were more localized, suggesting a better load distribution. However, the trapezoidal thread designs in Figure 1 exhibited higher stress magnitudes in the implant than the square threads in Figure 2.

**Figure 3:**
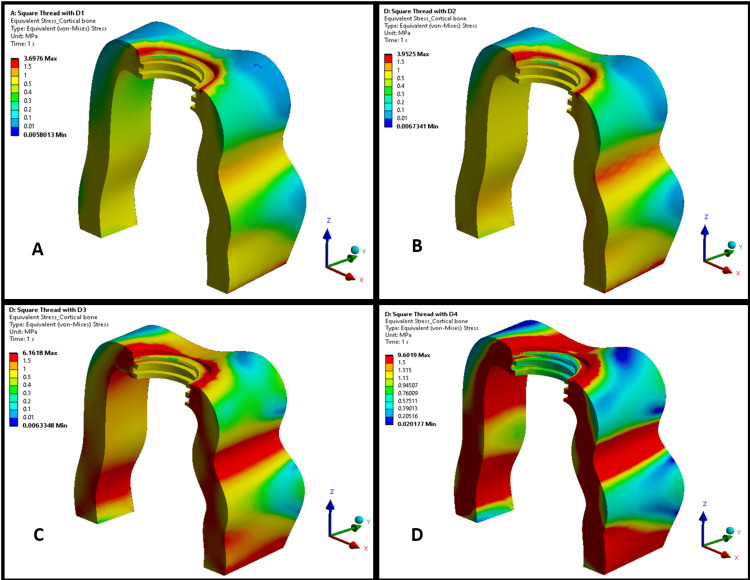
von Mises stress distribution in cortical bone in a square-threaded implant design in different bone densities: (A) D1 bone (dense cortical), (B) D2 bone (cortical/trabecular), (C) D3 bone (thin cortical/fine trabecular), and (D) D4 bone (fine trabecular). Colors indicate von Mises stress in megapascals (MPa): blue (low stresses), green (moderate), yellow (high), and red (critical). Arrows denote anatomical/loading directions. This figure is generated using Ansys software for finite element modeling of stress distribution.

**Figure 4:**
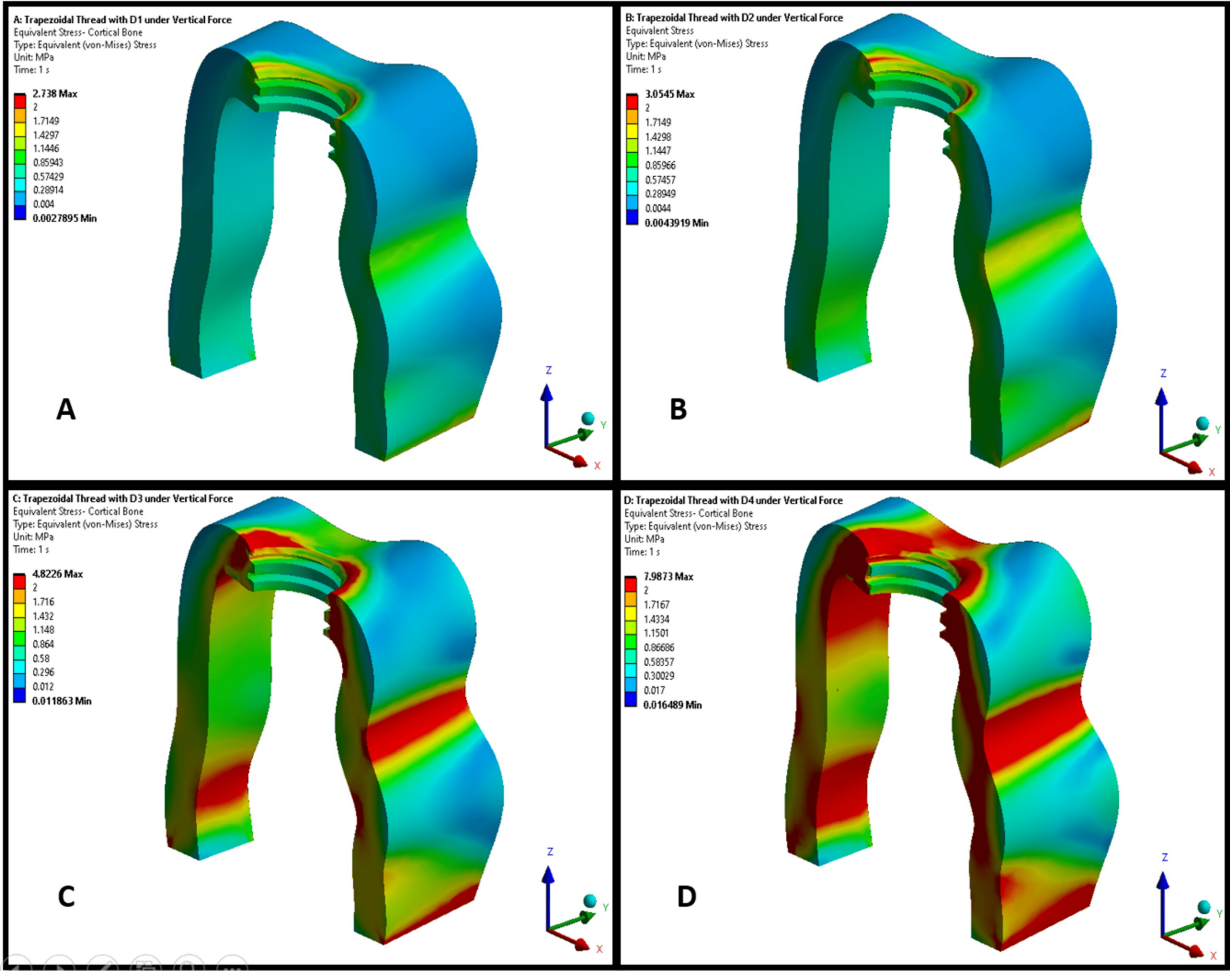
von Mises stress distribution in cortical bone in a trapezoidal-threaded implant design in different bone densities: (A) D1 bone (dense cortical), (B) D2 bone (cortical/trabecular), (C) D3 bone (thin cortical/fine trabecular), and (D) D4 bone (fine trabecular). Colors indicate von Mises stress in megapascals (MPa): blue (low stresses), green (moderate), yellow (high), and red (critical). Arrows denote anatomical/loading directions. This figure is generated using Ansys software for finite element modeling of stress distribution.

Figures 3, 4 illustrate the maximum von Mises stress distributions in the cortical bone for implants with square thread and trapezoidal thread designs, respectively, across bone densities D1-D4. Both figures demonstrate a trend of increasing stress in the cortical bone as bone density decreased from D1 to D4, with stress concentrations primarily observed at the bone-implant interface, particularly around the thread contact zones. In lower-density bones (D3 and D4), the stress patterns were more diffuse, indicating a broader load distribution owing to reduced bone stiffness. In higher-density bones (D1 and D2), the stresses were more localized, suggesting a more effective load transfer. However, square thread designs (Figure 3) consistently exhibited higher cortical bone stresses than trapezoidal thread designs (Figure 4) across all densities. This indicated that trapezoidal threads transferred less stress to the cortical bone, potentially reducing the risk of bone damage.

**Figure 5:**
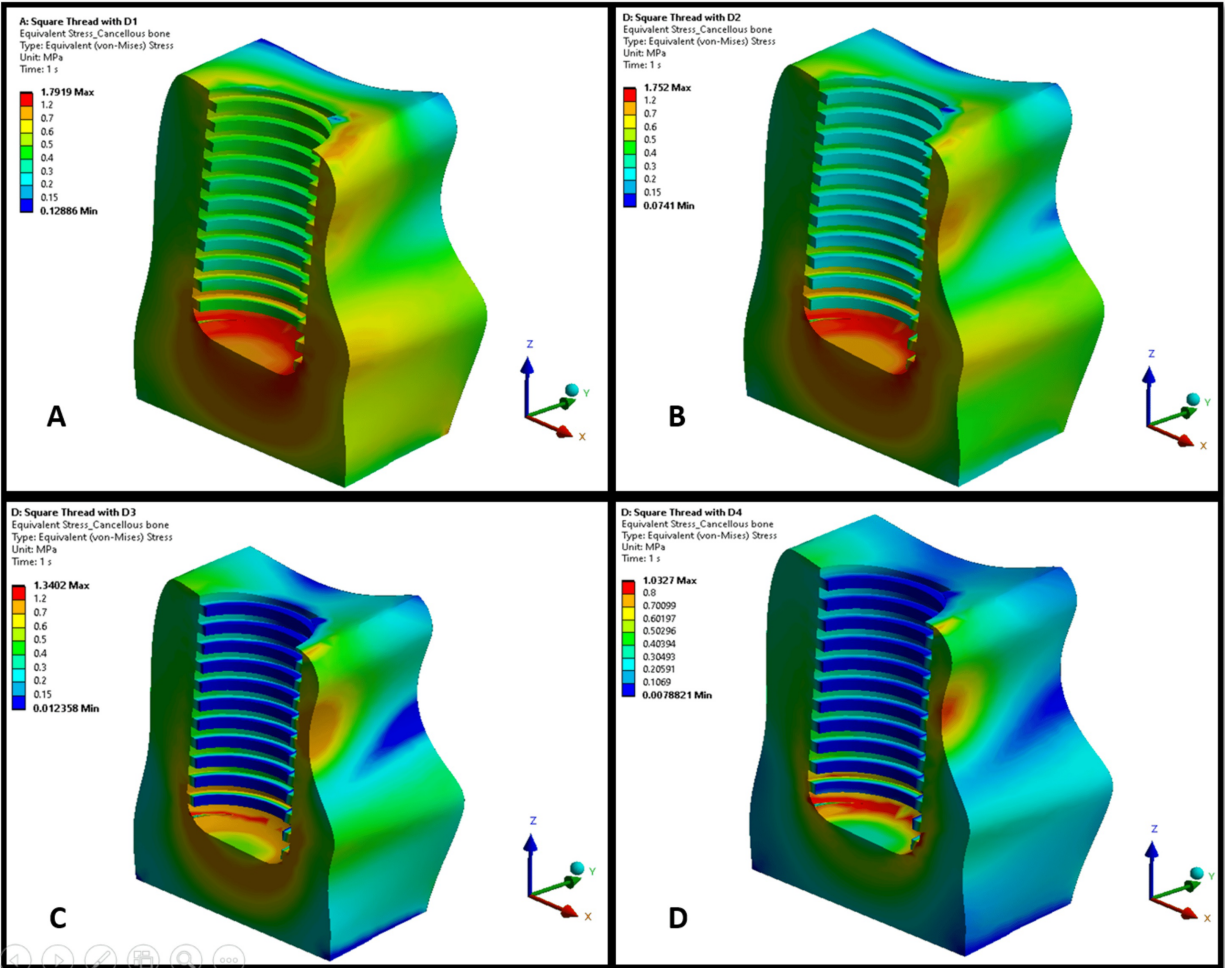
von Mises stress distribution in cancellous bone in a square-threaded implant design in different bone densities: (A) D1 bone (dense cortical), (B) D2 bone (cortical/trabecular), (C) D3 bone (thin cortical/fine trabecular), and (D) D4 bone (fine trabecular). Colors indicate von Mises stress in megapascals (MPa): blue (low stresses), green (moderate), yellow (high), and red (critical). Arrows denote anatomical/loading directions. This figure is generated using Ansys software for finite element modeling of stress distribution.

**Figure 6:**
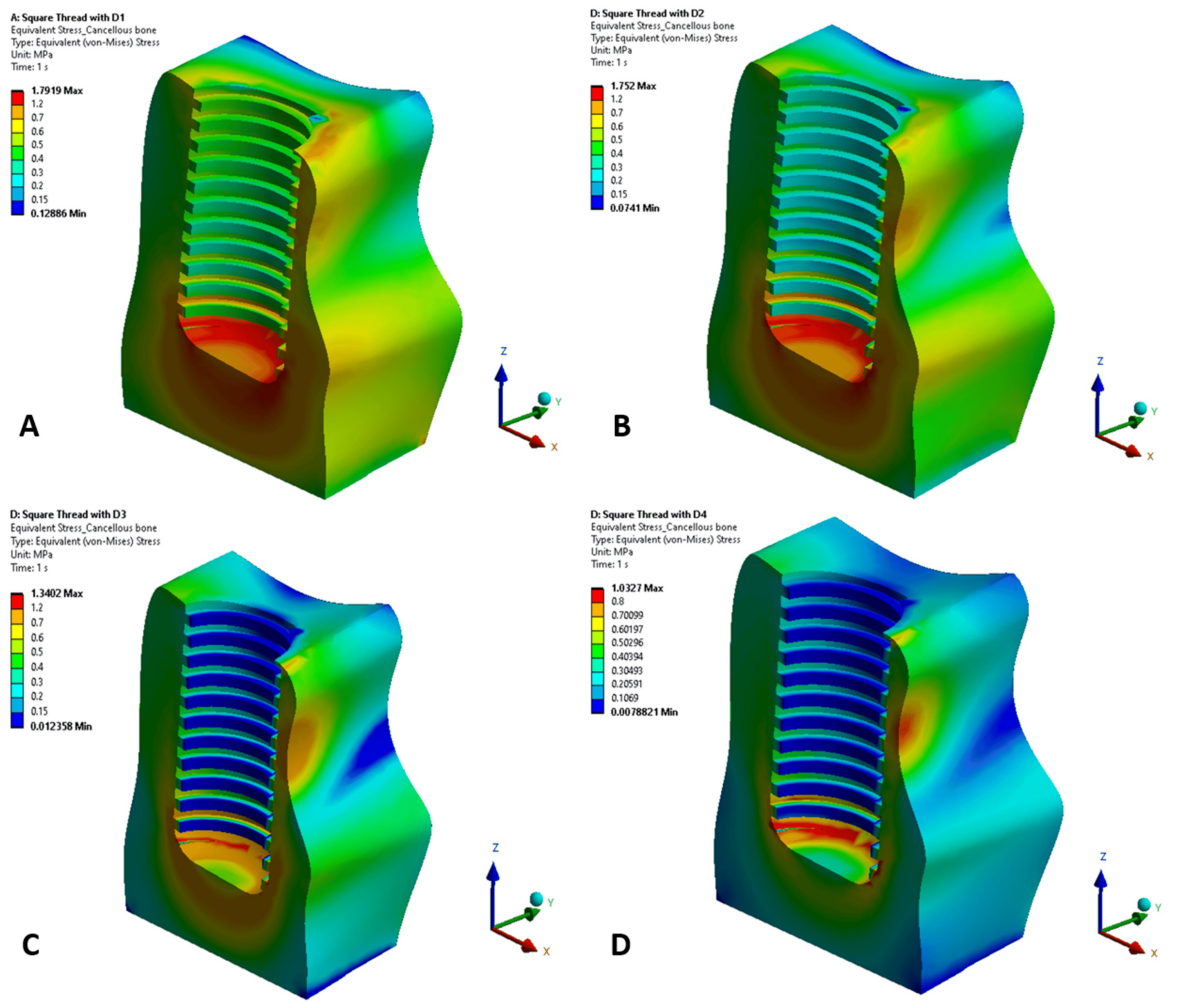
von Mises stress distribution in cancellous bone in a trapezoidal-threaded implant design in different bone densities: (A) D1 bone (dense cortical), (B) D2 bone (cortical/trabecular), (C) D3 bone (thin cortical/fine trabecular), and (D) D4 bone (fine trabecular). Colors indicate von Mises stress in megapascals (MPa): blue (low stresses), green (moderate), yellow (high), and red (critical). Arrows denote anatomical/loading directions. This figure is generated using Ansys software for finite element modeling of stress distribution.

Figures 5, 6 illustrate the maximum von Mises stress distributions in cancellous bone for implants with square thread and trapezoidal thread designs, respectively, across bone densities D1-D4. Both figures show a decreasing trend in cancellous bone stress as bone density decreased from D1 to D4, with stress concentrations primarily located at the bone-implant interface, particularly around the thread contact zones. In higher-density bones (D1 and D2), stresses were more localized, reflecting better load-bearing capacity, while in lower-density bones (D3 and D4), stress patterns were more diffuse owing to reduced bone stiffness. Trapezoidal thread designs exhibited higher cancellous bone stresses in higher-density bones, but converged with square thread stresses in lower-density bones. This suggests that trapezoidal threads transfer greater stress to cancellous bone in denser conditions, whereas square threads distribute stresses more evenly across densities.

## Discussion

The FEA conducted in this study provided valuable insights into the biomechanical behavior of dental implants with square and trapezoidal thread designs across varying bone densities (D1-D4). The study revealed that the stress distribution patterns in the implant body, cortical bone, and cancellous bone were significantly influenced by both the thread design and bone density. Specifically, trapezoidal thread designs consistently generated higher stresses in the implant body than square thread designs, particularly as the bone density decreased. This observation can be attributed to the geometric characteristics of the trapezoidal threads, which have a wider thread face and a more angled profile, leading to an increased stress concentration at the thread crests and roots. The broader contact area of the trapezoidal threads likely resulted in greater load transfer to the implant, reducing the distribution of forces to the surrounding bone. A previous study by Geng et al. [11] similarly reported that trapezoidal thread designs tend to concentrate stress within the implant because of their geometry, which may amplify mechanical strain under axial loading conditions. As reported by a previous study, the nature of the stress conveyed to the osseous tissue is contingent upon the configuration of the thread, and alterations in the angles of the thread surface have been shown to induce variations in the type of stress experienced by the bone in proximity to the implant [4]. Sadr and Vahid Pakdel [12] reported increased von Mises stresses with a trapezoidal thread design at higher thread angles. In contrast, Eraslan and Inan [13] suggested that square threads distributed von Mises stresses in a manner similar to V-shaped, buttress, and reverse buttress thread designs.

In the cortical bone, the square thread designs exhibited higher stress levels than the trapezoidal threads across all bone densities. This finding likely stems from the square threads’ ability to engage more effectively with the cortical bone owing to their perpendicular thread flanks, which enhance the mechanical interlocking and load transfer to the denser cortical layer. As bone density decreased, stress in the cortical bone increased for both thread designs; however, the effect was more pronounced with square threads, particularly in lower-density bones (D3 and D4). This can be explained by the reduced structural support in less dense bones, which forces the cortical layer to bear a greater proportion of the applied loads. A previous study by Eraslan and Inan [13] supports this observation, noting that square threads tend to transfer higher stresses to the cortical bone because of their design, which maximizes the contact area and mechanical stability. Alresheedi et al. [6] suggested that trapezoidal threads reduce cortical bone stress by distributing loads more evenly to the cancellous bone, particularly in denser bone types, which partially aligns with the lower cortical stresses observed with trapezoidal threads in this study. Lee et al. [14] determined that the trapezoidal thread exhibited the lowest maximum principal stresses, which was attributed to its superior bone contact area relative to other thread configurations.

For the cancellous bone, the stress distribution showed an opposite trend, with the stress decreasing as the bone density decreased for both thread designs. Trapezoidal threads induced higher stress in the cancellous bone under higher-density conditions (D1 and D2); however, the stress converged with that of square threads in lower-density bones (D3 and D4). This pattern can be attributed to the role of the cancellous bone as a secondary load-bearing structure, where its lower stiffness in the D3 and D4 bones results in reduced stress transmission [15]. Higher cancellous bone stress with trapezoidal threads in denser bones likely arises from their angled thread geometry, which facilitates deeper penetration and greater load transfer to the cancellous core. This is supported by the study of Chun et al. [16], who found that trapezoidal threads enhance load distribution to the cancellous bone in high-density conditions because of their ability to engage a larger volume of bone. Conversely, Huang et al. [17] indicated that square threads may distribute stresses more uniformly across both cortical and cancellous bones, reducing peak stresses in the cancellous bone, which aligns with the findings in lower-density conditions in this study.

The stress concentration patterns observed across the implant, cortical bone, and cancellous bone were primarily located at the bone-implant interface, particularly around the thread contact zones. In higher-density bones, the stresses were more localized, reflecting the ability of the bone to resist deformation effectively. In contrast, lower-density bones exhibited more diffuse stress patterns, indicating greater strain due to reduced structural support. This trend was consistent across both thread designs but was more pronounced with trapezoidal threads in the implants and square threads in the cortical bone. Localized stress in denser bones suggests efficient load transfer, whereas diffuse patterns in less dense bones highlight the challenges of achieving implant stability in compromised bone quality. These findings are corroborated by Sevimay et al. [15], who noted that stress distribution becomes more widespread in lower-density bones because of decreased mechanical support, which increases the risk of micromotion of the implant. However, Tada et al. [18] argued that thread design has a limited impact on stress distribution in very low-density bones, as the mechanical properties of the bone dominate the biomechanical response.

The differences in stress distribution between the square and trapezoidal thread designs have significant implications for implant design and clinical practice. Square threads appear to offer a biomechanical advantage by reducing implant stress, which may enhance the longevity of implants by minimizing fatigue-related failure [19]. However, their tendency to transfer higher stress to the cortical bone could increase the risk of bone resorption or microfractures, particularly in patients with compromised bone densities. Trapezoidal threads induce higher implant stresses, reduce cortical bone stress, and potentially preserve bone integrity under denser conditions [16]. Higher cancellous bone stress in denser bones may also improve primary stability by increasing bone volume [15]. These findings suggest that thread design selection should be tailored to the patient’s bone quality, with square threads potentially better suited for denser bones (D1 and D2) and trapezoidal threads offering advantages in lower-density bones (D3 and D4) to optimize load distribution and implant stability.

Clinical implications

The results of this study have important clinical implications for dental implants. The choice of thread design can significantly influence the biomechanical performance of implants, particularly in patients with varying bone densities. Square thread designs may be preferred in patients with high-density bone (D1 and D2) to minimize implant stress and enhance mechanical durability, provided that cortical bone integrity is monitored to prevent overloading. Trapezoidal threads, with their lower cortical bone stresses and higher cancellous bone engagement in denser conditions, may be more suitable for patients with moderate to low bone density (D3 and D4), in whom preserving the bone structure and achieving primary stability are critical. Clinicians should consider preoperative bone density assessments, such as those obtained from CBCT scans, to guide thread design selection and optimize implant success. Additionally, the observed stress patterns underscore the importance of precise implant placement and load application to minimize stress concentrations and reduce the risk of bone damage or implant failure.

Limitations

This study had several limitations that should be considered when interpreting the results. FEA is based on simplified assumptions, including homogeneous, linearly elastic, and isotropic material properties, which may not fully reflect the complex anisotropic behavior of biological tissues, such as bone [20]. This study utilized a single static load of 100 N applied axially, which does not account for the dynamic or multidirectional loading conditions encountered in clinical practice. The bone model was limited to a single mandibular first molar region, and variations in bone anatomy or implant positioning have not been explored. Additionally, this study did not account for biological factors, such as bone remodeling, osseointegration, or soft tissue interactions, which could influence long-term implant performance. Future studies should incorporate dynamic loading, patient-specific bone models, and biological factors to provide a more comprehensive understanding of implant biomechanics.

## Conclusions

The study concluded that thread design and bone density significantly influenced stress distribution in the implant, cortical bone, and cancellous bone. Trapezoidal thread designs consistently produced higher stresses in the implant body than square thread designs, particularly in lower-density bones, whereas square threads transferred greater stresses to the cortical bone across all bone densities. In the cancellous bone, trapezoidal threads generated higher stresses under denser conditions, but the stresses converged with those of square threads in lower-density bones. Stress concentrations were predominantly observed at the bone-implant interface, with localized patterns in denser bones and more diffuse distributions in less dense bones. These findings highlight the importance of selecting thread designs based on bone quality to optimize load distribution and enhance implant stability, with square threads potentially better suited for denser bones and trapezoidal threads offering advantages in lower-density conditions.
